# Time-series metabolomic profiling of SARS-CoV-2 infection: Possible prognostic biomarkers in patients in the ICU by ¹H-NMR analysis

**DOI:** 10.1371/journal.pone.0327244

**Published:** 2025-07-03

**Authors:** Emir Matpan, Ahmet Tarık Baykal, Lütfi Telci, Türker Kundak, Mustafa Serteser

**Affiliations:** 1 School of Medicine, Department of Medical Biochemistry, Acibadem Mehmet Ali Aydinlar University, Istanbul, Turkey; 2 Acibadem Labmed Clinical Laboratories, Istanbul, Turkey; 3 Acibadem International Hospital, Divisions of Anesthesiology and Reanimation, and General Intensive Care, Istanbul, Turkey; 4 Division of Internal Medicine, Acibadem International Hospital, Istanbul, Turkey; University of California Riverside, UNITED STATES OF AMERICA

## Abstract

The global impact of SARS-CoV-2, which causes COVID-19, remains significant, being intensified by the emergence of variants. Comprehensive metabolomic studies aimed to elucidate the distinctive metabolic footprint of the virus. For critically ill patients with COVID-19 in the intensive care unit (ICU), longitudinal monitoring based on their prognosis is crucial to optimize treatment outcomes. This study retrospectively investigated the temporal changes in the metabolomic profiles of patients admitted to the ICU with COVID-19, who were categorized into three prognostic groups: healthy discharged (HD), polyneuropathic syndrome (PS), and Exitus. In total, 32 serum samples collected in April 2020 at regular intervals (four samples per patient) and stored at −80°C, were analyzed using proton nuclear magnetic resonance (^1^H-NMR) spectroscopy. Significant (p < 0.05) prognostic changes in creatine and tyrosine levels were revealed by two-way analysis of variance (ANOVA) and ANOVA–simultaneous component analysis (ASCA). Furthermore, supervised random forest analysis demonstrated excellent group prediction with a 21.9% out-of-bag error rate based on prognosis. Specifically, creatine levels were highest in the PS group, whereas tyrosine levels were highest in the Exitus group. However, no metabolite displayed significant changes over time. In addition, metabolic pathway analysis using the Kyoto Encyclopedia of Genes and Genomes database indicated that the most significantly impacted pathway (p < 0.05) across different prognostic groups was “phenylalanine, tyrosine and tryptophan biosynthesis.” This preliminary study emphasizes the need for time-series analysis of samples from unvaccinated patients with varying prognoses, providing valuable insights into the metabolic impact of COVID-19.

## Introduction

COVID-19, caused by SARS-CoV-2 infection, has led to numerous hospitalizations and millions of deaths globally since the initial emergence of the virus [1]. Because of the strong mutative capability of the virus, it continues to cause illness ranging from mild symptoms such as fever, myalgia, and cough to multisystem disorders requiring intensive care unit (ICU) admission and even death [2]. Beyond the acute phase, many patients experience post-acute COVID-19 syndrome (PACS; symptoms persist for more than 4 weeks) characterized by prolonged, systemic complications affecting multiple organ systems and metabolic pathways. Despite ongoing research, there remain substantial gaps in the mechanistic understanding of PACS and the intricate systemic dysfunction leading to prolonged symptoms and lasting damage [3]. Despite efforts to identify effective therapeutic management strategies, accurate risk categorization for patients following SARS-CoV-2 infection remains challenging because of the complex nature of COVID-19, which causes rapid phenoconversion characterized by the transition from a normal or healthy state to a pathological condition. This phenomenon is often associated with changes in the levels of various metabolic biomarkers, which can be precisely analyzed in relation to the disease state and severity through metabolomics research [4]. Various analytical metodologies, including liquid chromatography–mass spectrometry (LC-MS) [4], gas chromatography–mass spectrometry (GC-MS) [5], and quantitative nuclear magnetic resonance (NMR) spectroscopy [6], have been employed to investigate metabolic alterations in COVID-19 patients and measure biochemical components in multiple pathways associated with the clinical signs, symptoms, and whole-body responses to the disease [7].

However, studies focusing on prognostic and real-time disease stratification among only critical cases requiring ICU admission, namely personalized metabolic screening measurements to assess metabolite changes caused by SARS-CoV-2 in ICU-treated patients with different prognoses, are limited. Approximately 15–25% of hospitalized patients with COVID-19 require ICU admission, and one-third of these patients die during treatment, thereby straining the limited resources of specialized facilities globally [8]. Additionally, the heterogeneity of patients with COVID-19 and the urgent need to provide the right treatment further complicate clinical decision-making regarding medication, intervention, and resource planning in ICUs [9]. In this context, measuring metabolic biomarker levels could have a crucial role in assessing biological processes before the exacerbation of clinical conditions [8], making them potentially valuable tools for the efficient management of infectious diseases such as COVID-19 at the ICU level.

Previous metabolomic studies using proton NMR (^1^H-NMR) have identified several metabolic disturbances, including altered kynurenine and arginine pathways which are linked to immune response modulation and inflammation [10], disrupted amino acid profiles [11], and elevated levels of ketone bodies and markers of liver and kidney dysfunction [12]. Notably, changes in metabolites such as tryptophan, tyrosine, lysine, glutamine, phenylalanine, creatine and creatinine have been associated with immune response, inflammation, and organ damage in severe COVID-19 cases in some other studies [13,14].

The present study utilized 1H-NMR to investigate metabolic changes with high accuracy and sensitivity using serum samples from patients admitted to the ICU because of SARS-CoV-2 infection with different prognostic outcomes. Serum samples were collected at specific intervals to capture dynamic metabolic changes over time, providing insight into disease progression and potential biomarkers for prognosis. By analyzing time-based metabolic fingerprint changes, we aimed to identify metabolites associated with severe disease progression and develop preliminary data to support targeted therapeutic strategies. Additionally, our preliminary study evaluated the impact of prognostically and temporally distinct serum metabolites on metabolic pathways, identifying the associated biochemical processes.

## Materials and methods

### Study design and participants

In this study, serum samples (n = 32) collected retrospectively in April 2020 at regular intervals (0 (April 10, 2020), 1st (April 13, 2020), 2nd (April 17, 2020), and 3rd (April 20, 2020) time points, giving four samples per patient) and preserved at −80°C were subjected to metabolomic analysis by ^1^H-NMR. The samples were collected from patients (n = 8, four men and four women; 48–77 years old) who had been admitted to the ICU to receive supportive care for critical COVID-19 because of worsening conditions who experienced different prognostic outcomes [healthy discharged (HD, n = 4), polyneuropathic syndrome (PS, n = 2), and Exitus (n = 2)]. Additionally, the condition of all included patients was classified as clinically critical based on a WHO Ordinal Scale for Clinical Improvement (OSCI) [15] score of 5–8. The serum samples from patients were accessed on October 9, 2023, following the ethical committee approval obtained on October 6, 2023. Necessary arrangements and quality controls were conducted subsequently to facilitate metabolomic analyses for research purposes.

All patients had a positive confirmation of SARS-CoV-2 infection by RT-qPCR (CFX96 Touch Real-Time PCR Detection System, Bio-Rad, Hercules, CA, USA; Bio-Speedy^®^ COVID-19/Flu RT-qPCR Kit, Bioeksen Molecular Diagnostics, Istanbul, Turkey) using nasal swabs (vNAT^®^ Transfer Tube, Bioeksen Molecular Diagnostics). Prognostic groupings were based on patients’ conditions at the end of ICU treatment. For the PS and Exitus groups, considerations such as the limited number of available patient samples, appropriate collection times, sufficient sample quantity for analysis, and the value of the samples were evaluated. Consequently, 16 samples collected from 4 patients at 4 different time points were included in study. Similarly, the HD group comprised 16 samples collected at the same time points from 4 different patients matched for age and sex. The HD group samples, collected in real-time before the onset of the pandemic (without vaccination and multiple interferences), were considered valuable samples for determining the specific metabolic effects and metabolite changes in the serum samples of patients with critical COVID-19. Therefore, the HD group was accepted as the control group in this preliminary study but not regarded as a healthy control group. Additionally, the age, sex, ICU stay, and prognostic status of the patients included in the study are presented in (Table 1).

**Table 1:** Demographics of the patients included in this study. Age and ICU stay data are presented as mean ± standard deviation.

Prognosis	Number of Patients	Number of Samples	Age (Years)	Sex	ICU Stay (Days)
**Healthy Discharged**	4	16	61.75 ± 11.03	2 M/2 F	16.5 ± 3.5
**Polyneuropathic Syndrome**	2	8	63 ± 12.73	2 M	102 ± 53.7
**Exitus**	2	8	70.5 ± 9.2	2 F	24 ± 1.4

This study was conducted according to the guidelines of the Declaration of Helsinki and approved by the Acıbadem Healthcare Institutions Medical Research Ethics Committee (Confirmation number: 2023-15/523), and informed consent was obtained from all patients at the beginning of the sample collection in April 2020. In addition, patient information was kept confidential, taking patient privacy into consideration.

### Sample preparation for ^1^H-NMR spectroscopy

All patient samples, previously stored at −80°C, were thawed at 4°C for 1 hour prior to analysis. The samples were briefly vortexed and then centrifuged at 14,000 × g for 5 minutes at 4°C. The resulting supernatants were subsequently collected for further analysis. Each sample was prepared by adding a commercially prepared pH 7.4 sodium phosphate buffer (0.9% NaCl and 0.1% TSP in D₂O) (Bruker, Billerica, MA, USA) and serum at a 1:1 ratio, resulting in a final volume of 600 µL for each sample. Samples were then manually shaken gently for 1 min before being transferred into 5-mm SampleJet™ NMR tubes.

### Sample processing and quantitative ^1^H-NMR spectroscopy

All NMR spectroscopic analyses were performed on a 600 MHz Bruker Avance IVDr system (Bruker) equipped with a 5-mm BBI probe and fitted with the Bruker SampleJet™ robot cooling system set to 5°C. All methods were validated for COVID-19 samples based on previous research [16], and the required quantitative calibration was completed prior to starting the analysis. The B.I. BioBankQC™ module (Bruker) was used for quality control, and the B.I. QUANT-PS™ module (Bruker) was used for targeted metabolite quantification, providing automated data processing and acquisition (including acetic acid, acetoacetic acid, acetone, citric acid, creatine, creatinine, formic acid, glucose, d-3-hydroxybutyric acid, lactic acid, pyruvic acid, threonine, etc.). One-dimensional (1D) ^1^H-NMR spectra were acquired using Bruker’s standard NMR software TopSpin (version 3.6.2) at 300 K. The standard 1D NMR experiment with solvent suppression was applied using Bruker’s automated suppression protocols integrated into the B.I. QUANT-PS™ module with 32 scans with a total experiment time of 4 min. First, a nuclear Overhauser effect spectroscopy (NOESY) experiment was applied to control the NMR spectrum quality (using B.I. BioBankQC™) and enable the quantification of metabolites (B.I. BioBankQuant-PS™). In addition, a Carr–Purcell–Meiboom–Gill (CPMG) experiment was conducted to filter out the macromolecular signals. Statistical total correlation spectroscopy was also performed for each metabolite to analyze the signal patterns based on previous research [17]. The observed signal patterns related to the metabolites were integrated and correlated to the IVDr measurements to validate the quantification. Furthermore, the NMR metabolites were quantified using the external reference based on ERETIC (Electronic Reference To Access In Vivo Concentrations) calibration [18]. Calibration transfer to each measurement was provided by the PULCON principle defined in the reports [19]. An artificial reference peak, corresponding to a concentration of 10 mM, was added to the solvent-suppressed 1D NMR spectrum in a region free of metabolite signals automatically. Quantitative calibrations were then obtained and validated using a dedicated reference sample with known compound concentrations adhering to B.I. Methods QC procedures. All spectral data acquisition and processing were fully automated and standardized based on Bruker’s protocols, ensuring reproducibility and consistency across all samples. The preparation of serum samples and NMR protocols followed the B.I.-embedded standard operating procedures (SOP) as outlined in the study by Dona et al. [20].

### Statistical analysis and data interpretation

Statistical analysis was performed using the online comprehensive tool MetaboAnalyst (version 6.0). Missing values were managed via replacement by the limit of detection method (1/5th of the positive value of each variable). All spectra were baseline-corrected and normalized via logarithmic data transformation (base 10) with autoscaling (mean-centered and divided by the standard deviation of each variable). Both univariate and multivariate analyses were applied. Unsupervised interactive principal component analysis (PCA) using different colors or shapes based on selected metadata in 2D and 3D plots and supervised orthogonal partial least squares discriminant analysis (OPLS-DA) were performed. The data distribution was analyzed using the Shapiro–Wilk test, and differences across experimental groups were investigated using the Mann–Whitney U test and Kruskal–Wallis test.

Two-way analysis of variance (ANOVA) with post-hoc Bonferroni correction was applied to observe the impacts of two independent variables (prognosis and time) and their potential interaction. Linear covariate analyses were also conducted to determine the extent of the impact of the metabolites with significantly different levels among the groups in two-way ANOVA. The degree of differences between the pairwise groups is presented in (Table 2). In addition, repeated-measures ANOVA–simultaneous component analysis (ASCA) was performed to identify the major patterns related to the two given factors and their interaction, resulting in the formation of scree plots based on the previously described algorithm [21]. In addition, the validation of ASCA was performed using a permutation test for both prognosis and time and for their interaction. Furthermore, supervised random forest analysis was performed for both prognosis and time, providing variable importance plots and mean decrease accuracy results of the related metabolites to evaluate the classification performance of the metabolomics. Metabolites found to have statistical significance (p < 0.05) in both two-way ANOVA and ASCA with F value >2.5 (Two-way ANOVA) [22] and well modeled in leverage/SPE scatter plots (ASCA) [23] were identified precisely in consideration of prognosis, time, and their interaction. Finally, metabolic pathway analysis using the Kyoto Encyclopedia of Genes and Genomes (KEGG) database was conducted among the HD, PS and Exitus groups to investigate the metabolic pathways most likely to be affected by the metabolites with significantly different levels in two-way ANOVA and ASCA. The KEGG results were defined depending on the significant metabolites between the different groups, and the affected pathways were also evaluated using impact value >0.1 and p < 0.05.

**Table 2:** Linear covariate analysis of prognostically significant metabolites. Presenting the effect sizes of metabolites that showed significant differences among prognostic groups in ICU-treated COVID-19 patients. Metabolites were included based on an F-value > 2.5 and a p-value < 0.05. Metabolites marked with a single asterisk (*) were significant in both two-way ANOVA and ASCA, whereas those marked with double asterisks (**) were significant only in two-way ANOVA. Positive effect sizes indicate an increase in metabolite concentration between the compared groups, while negative values indicate a decrease.

Metabolites	Exitus vs. Healthy Discharged	Polyneuropathic Syndrome vs. Healthy Discharged	F value	Adj. p value
Creatine*	1.148	1.516	8.306	0.007
Tyrosine*	1.468	1.141	7.917	0.007
Formic acid**	0.782	1.545	7.422	0.008
Phenylalanine**	1.429	0.793	6.477	0.015
**Methionine**	0.998	1.301	6.170	0.016
**Ethanol**	1.089	1.154	5.684	0.019
**Acetone**	(-) 0.842	0.741	5.670	0.019
**Pyruvic acid**	0.745	1.311	5.488	0.020
**Acetic acid**	1.184	0.806	4.790	0.033
**Threonine**	(-) 1.156	(-) 0.846	4.735	0.033

## Results

### ^1^H-NMR–based metabolic differences among the HD, PS, and exitus groups

To investigate the specific metabolic changes induced by SARS-CoV-2 infection in patients with critical COVID-19 treated in the ICU, 32 real-time collected serum samples were collected from eight different patients. Based on their outcomes during treatment and follow-up in the ICU, the HD, PS, and Exitus groups included four, two, and two patients, respectively.

B.I. QUANT-PS™ analysis was performed to quantify the serum concentrations of 41 different metabolites, including trimethylamine-N-oxide, amino acids and derivatives, carboxylic acids, essential nutrients, keto acids and derivatives, carbohydrates and derivatives, and dimethylsulfone as a sulfone, defining them in mmol/L units.

**Fig 1:**
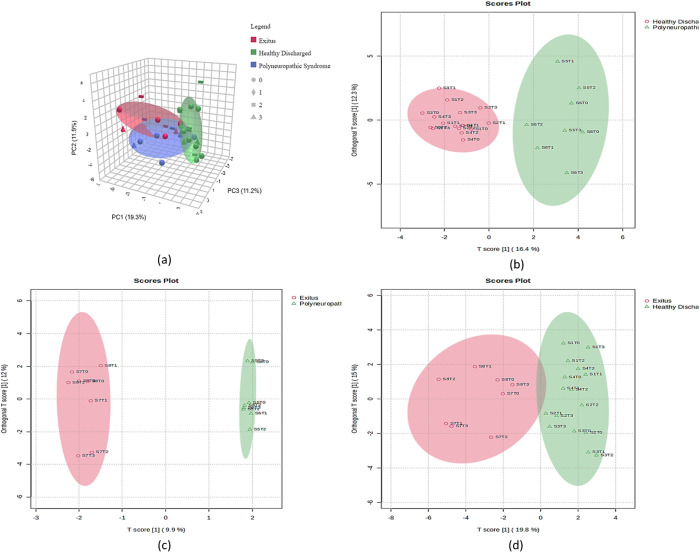
Multivariate analysis of prognostic groups in ICU COVID-19 patients over time. (a) An iPCA 3D score plot is presented in the middle of the diagram (classified by prognostic group by color and sample collection time by shape), with PC1 explaining the highest variance, followed by PC2 and PC3 (total variance: 42.4%). (b, c, d) 2D OPLS-DA score plots providing better discrimination between different prognostic groups (HD, PS, and Exitus) according to the collection times of the samples in patients with COVID-19 treated in the ICU.

Prior to this, a discriminating metabolite profile was formed using the interactive principal component analysis (iPCA) 3D scores plot consisting of both prognosis and time, in which principal components 1, 2, and 3 accounted for approximately 42.4% of the variation among groups (PC1: 19.3%, PC2: 11.9%, PC3: 11.2%). Additionally, gaining better separation between the different prognostic groups regarding the collection times of the samples in patients with COVID19 treated in the ICU, 2D OPLS-DA plots were also created, indicating clear separations among the pairwise compared groups (Fig 1).

**Fig 2:**
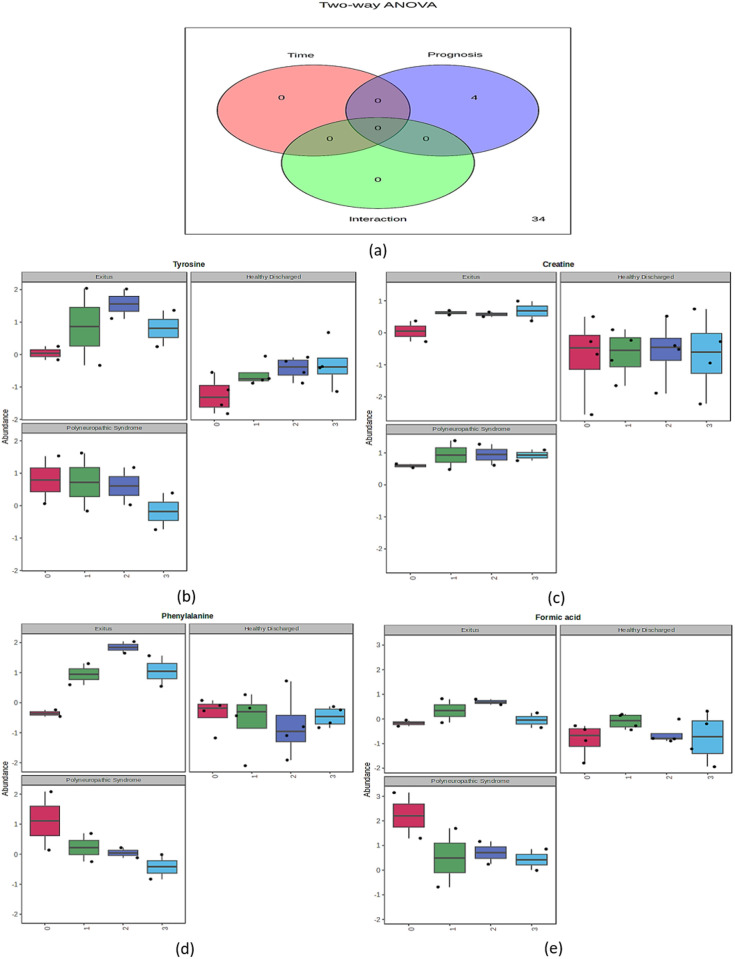
Temporal and prognostic variation of key metabolites in ICU COVID-19 patients. (a) Venn diagram of two-way ANOVA consisting of prognosis, time, and their interaction indicating the number of significant metabolites. (b, c, d, e) Box–whisker plots of four metabolites (tyrosine, creatine, phenylalanine, and formic acid respectively) with their change in relative abundance classified for prognosis (HD, Exitus, and PS) and collection times (0, 1st, 2nd and 3rd time points).

Following PCA and OPLS-DA (demonstrated clear distinctions between the groups), two-way ANOVA with post-hoc Bonferroni correction was conducted to investigate the metabolites leading to this separation among the compared groups. The analysis evaluated the effects of prognostic differences, sample collection times, and their interaction on the discrimination between samples. The results identified metabolites that potentially created significant separation among the groups. In two-way ANOVA, analytes that distinguished the groups were defined using the criteria of F > 2.5 [22] and p < 0.05. These criteria were applied separately to assess the effects of prognosis, the collection time, and their interaction. The detailed results are presented in (Table 3). Specifically, tyrosine, creatine, formic acid, and phenylalanine had significance (p < 0.05) in differentiating the groups, being attributed solely to their effects on prognosis (Fig 2). In the time and interaction variable sets, two-way ANOVA did not identify any metabolites with significantly different levels among the groups. Box–whisker plots were created to present the changes in analytes with significantly different levels based on prognosis and the collection time (Fig 2) according to two-way ANOVA. Additionally, linear covariate analyses were conducted to assess the impact of these significantly different analytes as identified by two-way ANOVA, determining the effect sizes of these analytes among the groups (Table 2). Examination of the box–whisker plots and the degrees of difference based on covariate analyses indicated that creatine levels were elevated in both the PS (1.516) and Exitus groups (1.148) compared with that in the HD group. Tyrosine levels were also higher in the PS (1.468) and Exitus groups (1.141) than in the HD group. Formic acid levels were significantly higher in the PS group (1.545) than in the HD group, whereas a moderate increase was noted in the Exitus group (0.782). Meanwhile, phenylalanine levels were significantly higher in both the Exitus (1.429) and PS groups (0.793) than in the HD group. Specifically, the most notable differences were observed for in creatine and tyrosine levels, indicating their potential as biomarkers for disease progression in the ICU based on the results of two-way ANOVA and linear covariate analysis.

**Table 3:** Integrated two-way ANOVA and ASCA analysis of key prognostic and temporal metabolite changes. This table summarizes metabolites with significant abundance differences (p < 0.05) identified by both two-way ANOVA (F > 2.5) and ASCA (significant leverage/SPE ratio). Metabolites are categorized by prognosis, sampling time, and their interaction. Creatine and tyrosine (*), significant in both analyses, are highlighted as key markers distinguishing prognostic groups (HD, PS, and Exitus).

Metabolites	Two-way ANOVA with Post-Hoc Bonferroni Correction						Anova Simultaneous Component Analysis (ASCA)								
	Prognosis		Time		Interaction		Prognosis			Time			Interaction		
	F value	p value	F value	p value	F value	p value	Leverage	SPE	p value	Leverage	SPE	p value	Leverage	SPE	p value
Tyrosine*	12.41	**0.001**	1.36	0.28	0.98	0.47	0.089	0.57	**<0.05**						
Creatine*	9.5	**0.001**	0.21	0.88	0.06	1.00	0.088	1.36	**<0.05**						
Formic acid	11.68	**0.001**	0.56	0.65	1.66	0.18									
Phenylalanine	9.8	**0.001**	0.06	0.98	2.21	0.09									
Isoleucine										0.10	0.68	**<0.05**			
Lysine										0.09	2.69	**<0.05**			
Glycerol										0.17	1.59	**<0.05**			
Lactic acid													0.20	0.81	**<0.05**
Citric acid													0.14	4.02	**<0.05**
N-Dimethylglycine													0.13	1.66	**<0.05**
3-Hydroxybutyric acid													0.14	1.45	**<0.05**

### Identification of prognosis, time, and interaction-based significant patterns

**Fig 3:**
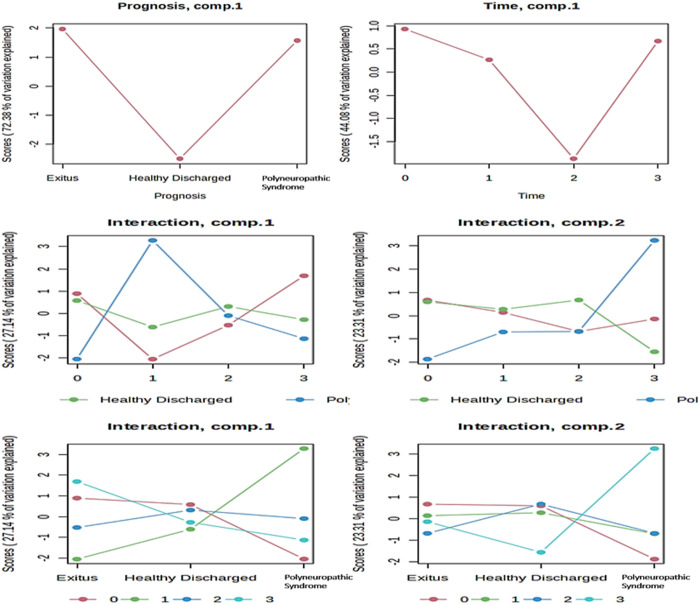
Principal patterns of metabolite variance across time and prognosis in ICU COVID-19 patients. Scree plots illustrating the major patterns of metabolite changes over time (0, 1st, 2nd, and 3rd time points) and across prognostic groups (HD, Exitus, and PS), along with their interaction. The scores in these plots represent the percentage of variance explained between the groups.

**Fig 4:**
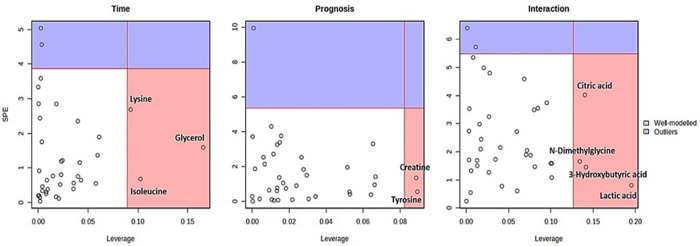
ASCA leverage/SPE analysis highlighting key metabolites associated with time, prognosis, and their interaction. The metabolites in the red area exhibit high loadings and align closely with the expression patterns delineated by the sub-models. By contrast, metabolites in the blue area (outliers) display expression patterns that deviate from these major patterns. The well-modeled metabolites in the red areas were specifically identified.

To systematically differentiate prognosis-based metabolite dynamics from the time dimension, ASCA was performed [24]. This statistical method separates variations of the whole dataset into parts devoted to the influences of different variables and their interactions [21]. ASCA was conducted to identify well-modeled components associated with prognosis, the collection time, and their interaction. Scree plots were used to graphically display the relationship between eigenvalues and factors, finding significant decreases in the magnitude of the eigenvalues and defining the number of factors to extract (Fig 3). The major patterns related to prognosis, the collection time and their interaction were presented in the score plots based on PC1 of the corresponding sub-models, indicating a decrease in the scores at the 2nd collection time and then a rapid increase at the 3rd collection time. The scores in the HD group (control) were significantly lower than those in the PS and Exitus groups for prognosis, and different patterns of changes were observed for the scores for the interaction (Fig 3). Moreover, the major trends associated with prognosis, time, and their interaction were delineated using leverage/squared prediction error (SPE) plots. Factors with a low SPE and higher leverage values were considered to significantly contribute to the related models (Fig 4).

**Fig 5:**
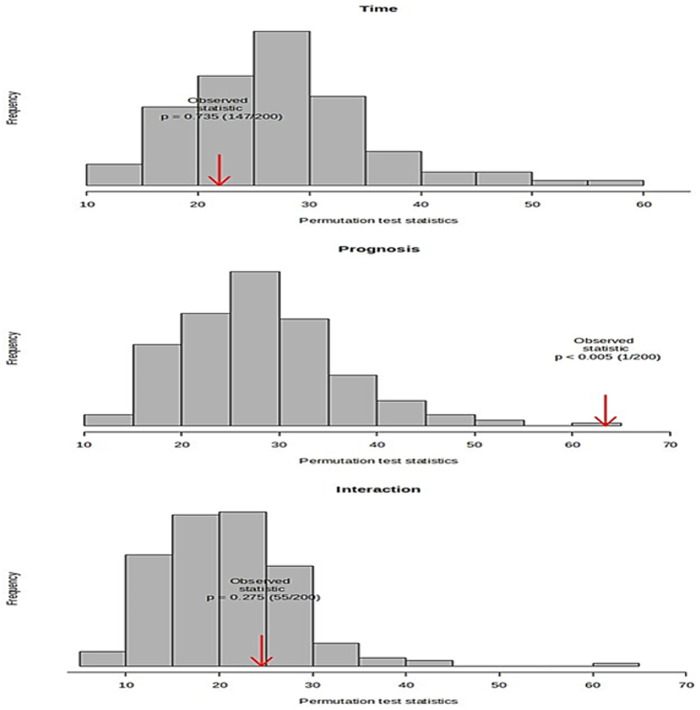
Permutation-based validation of ASCA models. Statistical significance was only observed for prognosis with p < 0.05.

The statistical power was evaluated using with a permutation test (200 times), which validated the model for prognosis, the collection time, and their interaction. However, based on the results of the validation model (Fig 5), although prognosis had a significant impact (p < 0.005) on the serum metabolome, the collection time (p = 0.735) and the interaction of these factors (p = 0.275) did not have significance. These results also denoted that prognosis and the collection time were independent factors, as indicated by their lack of interaction. Subsequent analysis identified creatine and tyrosine as the metabolites with significant effects on prognosis. Conversely, although lysine, isoleucine, and glycerol were identified as well-modeled analytes for the time variable and citric acid, N-dimethylglycine, 3-hydroxybutyric acid, and lactic acid were well-modeled analytes for the interaction in ASCA, they did not have significant effects on the serum metabolome because of the lack of statistical significance in the validation process using the permutation test.

**Fig 6:**
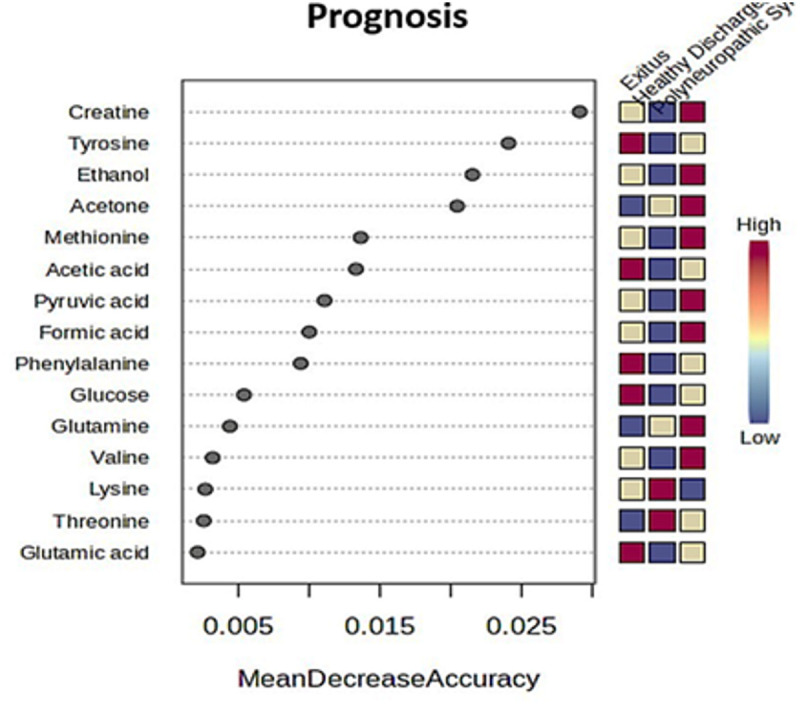
Random forest analysis. Variable importance plot highlighting prognostic metabolite predictors in ICU COVID-19 patients based on the mean decrease accuracy value. A higher value indicates the importance of that metabolite in predicting group with red color representing the higher importance, whereas blue color representing the lower importance in evaluation.

Furthermore, supervised random forest analysis was performed to identify the metabolites with the highest discriminatory impact among the three groups for prognosis using the mean decrease accuracy as the measure of model performance without each metabolite. A higher value indicated the importance of that metabolite in predicting groups (Fig 6). In addition, the random forest classification indicated excellent prediction of prognosis with an out-of-bag error rate of 21.9%, defining creatine and tyrosine as having the most influence on the classification.

### Significant metabolic pathway alterations

Datasets consisting of serum samples from each patient group in the ICU classified by prognosis (HD, PS, and Exitus) were submitted to pairwise analysis using the KEGG database on MetaboAnalyst (version 6.0). In this study, the primary metabolic pathways considered to be disrupted were identified through pairwise comparisons among the three prognostic groups and via evaluation of the impact of significant metabolites identified by two-way ANOVA or ASCA. These findings are presented in (Table 4). The identification and significance of the metabolic pathways presented in the table were determined using the criteria of p < 0.05 and impact > 0.1. However, only 5 of the 10 defined KEGG pathways (phenylalanine, tyrosine and tryptophan biosynthesis; glycine, serine and threonine metabolism; phenylalanine metabolism; arginine and proline metabolism; and tyrosine metabolism) were considered significant because of the impact of creatine or tyrosine, which exhibited overall statistical significance across all analyses in the study. Specifically, in comparison with the control group (HD), creatine was found to play a key role in the significant alterations observed in the “glycine, serine and threonine metabolism” and “arginine and proline metabolism” pathways. Meanwhile, tyrosine was a major contributor to changes in the “phenylalanine, tyrosine, and tryptophan biosynthesis,” “phenylalanine metabolism,” and “tyrosine metabolism” pathways, as presented in (Table 4). However, no significant metabolic pathway differences were found between the PS and Exitus group.

**Table 4:** KEGG pathway analysis of significantly altered metabolic pathways. This table presents KEGG pathway analysis based on metabolites identified as statistically significant (p < 0.05) through two-way ANOVA and ASCA, considering the effects of prognosis, sample collection time, and their interaction. However, no significant metabolic pathway alterations were found between the PS and Exitus groups. Therefore, no comparison between these groups was included in the table. Metabolic pathways significantly affected by differences in creatine or tyrosine levels across the different prognostic groups were also highlighted by double asterisks.

Pathway Name (KEGG)	Polyneuropathic Sendrom/ Healthy Discharged			Exitus/ Healthy Discharged		
	P-value	Metabolites	Impact	P-value	Metabolites	Impact
Glycine, serine and threonine metabolism**	< 0.001	Creatine N-Dimethylglycine	0.43	0.001	Creatine	0.36
Glyoxylate and dicarboxylate metabolism	< 0.001	Formic acid Citric acid	0.14	< 0.001	Formic acid Citric acid	0.14
Citrate cycle (TCA cycle)	< 0.001	Citric acid	0.23	0.002	Citric acid	0.22
Glycolysis/Gluconeogenesis	< 0.001	Lactic acid	0.13	< 0.001	Lactic acid	0.13
Arginine and proline metabolism**	< 0.001	Creatine	0.21	0.002	Creatine	0.20
Phenylalanine, tyrosine and tryptophan biosynthesis**	< 0.001	Tyrosine Phenylalanine	1.0	< 0.001	Tyrosine Phenylalanine	1.0
Phenylalanine metabolism**	< 0.001	Tyrosine Phenylalanine	0.36	< 0.001	Tyrosine Phenylalanine	0.36
Pyruvate metabolism	< 0.001	Lactic acid	0.27	< 0.001	Lactic acid	0.27
Tyrosine metabolism**	< 0.001	Tyrosine	0.14	0.002	Tyrosine	0.13
Butanoate metabolism				0.003	3-Hydroxybutyric acid	0.11
Galactose metabolism				0.013	Glycerol	0.04

Finally, phenylalanine and formic acid levels, which significantly differed among the prognostic groups only in two-way ANOVA, and citric acid, lactic acid, N-dimethylglycine, and 3-hydroxybutyric acid levels, which differed only based on ASCA analysis without passing validation tests, were also evaluated by KEGG analysis. Although some differences in metabolic pathways were observed across different prognostic groups based on these analytes, these findings were not considered statistically significant.

## Discussion

Although the impact of COVID-19 on mortality and morbidity has decreased compared with the early stages of the pandemic, variants arising from various mutations continue to lead to poor prognoses requiring ICU admission, particularly in immunocompromised patients, older adults, and patients with multiple comorbidities [25]. Since the onset of the pandemic, various integrated metabolomic studies have been conducted to investigate the fingerprint effects of SARS-CoV-2 on different human matrices (e.g., serum, plasma, urine) [9,26,27]. Despite these efforts yielding significant findings, studies assessing prognostic differences and real-time changes in patients with critical COVID-19 without interference from vaccination in the ICU setting remain rare.

In some studies, respiratory and systemic compromise, consisting of pneumonia-like symptoms, acute respiratory distress syndrome, or multiorgan failure, was observed in most critically ill patients admitted to the ICU for COVID-19, who had an average mortality rate of 41.6% (95% confidence interval = 34.0–49.7) [28,29]. To enhance the management and survival of critically ill patients with COVID-19 in ICU settings, it is essential to advance metabolomics studies, delineating the specific impact of SARS-CoV-2 on human plasma metabolites and facilitating the development of timely and effective interventions.

Hence, in the present preliminary study, the serum metabolic changes in critically ill patients admitted to the ICU for COVID-19, who had different prognoses, were investigated using serum samples collected at the beginning of the pandemic (April 2020) in a time-series manner. This approach minimized the impact of confounding factors such as vaccination or the impact of medications, thereby enhancing accuracy. The present study also identified the potential metabolic pathways involved in the pathophysiology of severe COVID-19, considering both different prognostic states and temporal changes in serum metabolite levels in patients in the ICU. More importantly, the obtained data illustrated that molecular changes at the metabolite level in patients in the ICU could be prospectively predictive of various prognostic outcomes.

Significant changes were observed in the levels of specific metabolites (creatine, tyrosine, formic acid, and phenylalanine) during the prognostic evaluation, indicating that prognostic differences in patients in the ICU could lead to diverse dysregulation in some specific metabolic pathways.

Creatine and tyrosine levels were higher in the PS and Exitus groups than in the HD group, as presented in (Table 2). Specifically, tyrosine levels were highest in the Exitus group, whereas creatine levels were found highest in the PS group. The highest elevation of creatine levels in the PS group could be explained by the widespread dysregulation of nitrogen metabolism and the long-term impact of renal dysfunction [28]. Additionally, the reversible conversion of phosphocreatine to creatine, primarily occurring in muscle cells and facilitated by creatine kinase, was revealed in previous studies to increase in response to severe infections and cellular damage [30]. This phenomenon was also consistent with the findings of our study, suggesting that during massive inflammation and increased energy demands, such as those observed in COVID-19, the conversion of phosphocreatine to creatine leads to a corresponding increase in serum creatine levels [31]. Moreover, the more pronounced increase in serum creatine levels in patients in the PS group than in those in the Exitus group might be attributable to the impairment of peripheral muscle stimulation, resulting in greater muscle degradation during ICU treatment. Another metabolomic study also evaluated the levels of creatine and phosphocreatine, which are markers of cerebral bioenergetics [32], in the corpus callosum tissue of post-COVID-19 patients exhibiting neurological symptoms such as PS, reporting that the tissue accumulation of these analytes did not significantly differ between patients and healthy controls. However, in our study, we identified significant elevations in serum metabolite levels in the PS and Exitus groups and significant alterations in some metabolic pathways (e.g., glycine, serine, and threonine metabolism, arginine and proline metabolism) [27] in pairwise group comparisons of prognosis based on the changes in serum creatine levels. These alterations were also found to be associated with more severe and significantly disrupted energy metabolism caused by COVID-19, leading to a massive inflammatory response (cytokine storm) [33], oxidative stress (impaired mitochondrial functions) [34] and organ dysfunction (especially in the liver and kidneys). Although some studies demonstrated an increase in creatine metabolite levels, similar to inflammatory markers such as CRP and IL-6 [33], in patients with COVID-19, our study indicated that fluctuating creatine levels in patients in the ICU could be predictive of different prognostic outcomes. Furthermore, a study comparing COVID-19–positive patients and their healthy controls did not identify differences in creatine levels between the groups, but important differences were observed between patients with severe COVID-19 and those with mild disease or healthy controls in another study [33]. This highlighted the importance of evaluating metabolite changes within critically ill patient groups and identifying different metabolites that could be used to monitor disease progression [14]. Additionally, it was also reported that patients who recover from severe COVID-19 often present with neurological sequelae and potentially experience multiorgan failure during hospitalization, indicating significant fluctuations in the plasma concentrations of certain amino acids [35] and their metabolites, such as reduced levels of alanine, tryptophan, serine, glutamine, and histidine or increased levels of phenylalanine, tyrosine, and creatine [14] similar with the present findings. This was because certain amino acids have a critical role in the immune system, cell regeneration, or tissue repair [36] and therefore, abnormalities in their metabolism could cause neurological deficits or multiorgan failure [37]. Thus, our study contributes to the literature by demonstrating significant changes in the levels of serum metabolites such as creatine, tyrosine, formic acid, and phenylalanine that can be utilized for prognostic differentiation and monitoring of the progression of critically ill patients with COVID-19 in the ICU.

Concerning tyrosine, an important precursor for the synthesis of neurotransmitters, thyroid hormones, melanin, and various proteins, previous studies identified a significant alteration in its plasma levels and metabolism in patients with severe COVID-19, suggesting a metabolic shift associated with disease progression [38,39], supporting the results of our study. Some metabolomic studies also demonstrated markedly elevated levels of tyrosine and other aromatic amino acids such as phenylalanine and tryptophan in patients with severe COVID-19, indicating systemic inflammation-related hepatic dysfunction or increased protein catabolism [31,40]. Although the findings of our study were similar to those of previous research, our work contributed to the literature from a novel perspective. Specifically, our study consisted exclusively of critically ill patients with COVID-19 (OSCI score = 5–8) and different prognoses who received treatment in the ICU, underscoring the importance of monitoring fluctuations in metabolite levels, such as creatine and tyrosine, during the treatment of such critically ill patients. Such surveillance could be beneficial for understanding the metabolic alterations that occur during critical infections, which might influence disease progression and outcomes and inform therapeutic strategies. Additionally, our study was specifically designed to assess samples from critically ill patients collected at the beginning of the pandemic to eliminate any potential interference from vaccine-related effects. This also included both prognostic and time-based assessments of these samples, differentiating this study from previous studies [27,41]. A targeted metabolomics analysis using ultra-performance liquid chromatography tandem mass spectrometry (UPLC-MS/MS) identified glycodeoxycholic acid, taurodeoxycholic acid, and tyrosine as metabolites with high predictive value for severe COVID-19 [39]. Consistent with these findings, the study reported elevated plasma tyrosine levels in patients with severe COVID-19 compared to healthy controls and those with mild disease. Similarly, our study demonstrated a significant increase in serum tyrosine levels in the poor prognostic groups (Exitus and PS) among critically ill COVID-19 patients in ICU, further supporting the prognostic relevance of tyrosine from a different perspective. Additionally, in the same study, when comparing the metabolic pathways affected in severe COVID-19 cases with those in mild cases, the pathway with the greatest impact was identified as ‘phenylalanine, tyrosine, and tryptophan biosynthesis,’ aligning with the metabolic alterations observed in our study [39]. In some other studies comparing patient groups during the recovery phase of acute COVID-19, the levels of both tyrosine and its precursor phenylalanine were elevated during the disease phase, but their levels decreased with recovery [31,41]. In our study, the levels of these aromatic metabolites were also elevated in the Exitus and PS groups compared with those in the control group. However, despite a similar proportional increase in tyrosine levels in both groups, the proportional increase in phenylalanine was significantly greater in the Exitus group (Table 2). This distinction could highlight the potential differential role of the phenylalanine/tyrosine ratio [40] in the surveillance of critically ill patients with COVID-19. Moreover, the findings of KEGG analysis in our study revealed that “phenylalanine, tyrosine and tryptophan biosynthesis” [27] and “phenylalanine metabolism” [31,42] which were significantly influenced by tyrosine and phenylalanine, exhibited significant alterations even in critically ill patients with COVID-19 with varying prognoses. These changes effectively differentiated the PS and Exitus groups from the HD group, leading to significant impact values (Table 4). Given the critical roles of these pathways in immune function [43], neurotransmitter production [44], and cellular stress responses [40], these alterations appeared to substantiate the conclusions of our study. More specifically, increased phenylalanine and tyrosine levels during sepsis or viral infections have been linked to immune system activation and increased cardiovascular disease risk, similar to that found in patients with severe COVID-19 [41,45]. Additionally, considering the synthesis of tyrosine-based neurotransmitters such as dopamine, epinephrine, and norepinephrine, it was noted that changes in the concentrations of tyrosine and its metabolites could significantly affect neurological function [31,46], highlighting a key underlying cause of the significant metabolite changes observed in our study. Aromatic amino acids have also been shown to activate B-cells, thereby stimulating the immune system during severe infections [45]. Therefore, the results of our study similarly explain the cause of the increased serum or plasma concentrations of these amino acids in severe infections. Recent studies also demonstrated that an increase in the phenylalanine/tyrosine ratio is indicative of disease severity and progression [40,46]. Although this ratio was not directly analyzed as a comparative parameter in our study, its evaluation indicated a greater increase in the PS group than in the Exitus group relative to the control group. Contrarily, a few studies presented findings contrary to those of our study. For instance, some research reported lower levels of tyrosine in the COVID-19 group than in the healthy control group regardless of disease severity [12]. Additionally, other research recorded reduced tyrosine levels alongside an increased phenylalanine/tyrosine ratio in patients with COVID-19 in the ICU [47] All of these findings further emphasize the need for more comprehensive time-series and prognostic studies to investigate the changes in the levels of aromatic amino acids and the phenylalanine/tyrosine ratio as distinguishing factors among critically ill patients with COVID-19 in the ICU.

The current literature features only a few studies assessing changes in formic acid levels in human urinary samples related to COVID-19 [26,48]. In one of these studies, it was suggested that urinary formic acid levels varied by age among patients with COVID-19 [26]. Additionally, the increase in urinary formic acid levels in patients with COVID-19 was attributed to the alterations in their bacterial flora in another study [48]. Furthermore, another metabolomic study reported elevated plasma formic acid levels in HIV-infected patients caused by the disruptions in the “glyoxylate and dicarboxylate metabolism” pathway associated with altered gut mucosal integrity, microbiome changes, and chronic inflammation [49]. In general, these studies indicated the potential metabolic and microbial factors contributing to formic acid level variations in viral infections such as COVID-19 and HIV, suggesting a broader connection among metabolism, immune responses, and microbial health in the context of viral diseases. Depending on our study, we observed elevated serum formic acid levels in both the PS and Exitus groups compared with those in the control group and disruptions in the ‘glyoxylate and dicarboxylate metabolism’, contributing new insights into the existing literature. In this preliminary study, formic acid levels were 2-fold higher in the PS group than in the Exitus group. This variation might be associated with several factors specific to the patients, including hypoxia levels, disruptions in acid–base homeostasis, and the increased synthesis of toxic organic acids such as formic acid. Finally, significant differences in formic acid levels were observed by two-way ANOVA but not by ASCA (evaluated as the leverage/SPE ratios) in terms of prognosis among the groups in this study. Therefore, based only on the present findings, the assessment of changes in serum formic acid levels cannot be considered fully reliable for differentiating prognostic groups of critically ill patients with COVID-19 in the ICU. These findings highlighted the potential metabolic differences among different patient groups, warranting the need for further investigations in more comprehensive studies.

In this study, lysine, isoleucine, and glycerol displayed well-modeled patterns in time-based evaluations only in ASCA based on the real-time changes in their serum concentrations in critically ill patients with COVID-19, whereas they could not be considered as significant metabolites because of the result of validation process using permutation testing (p = 0.735). Some studies revealed a correlation between these analytes and COVID-19 disease severity [50]. Specifically, isoleucine has been described as important in SARS-CoV-2 replication [50], with studies demonstrating that decreases in its plasma levels were associated with poor prognosis [12]. However, because time was not identified as a significant factor in the validation process of ASCA, the results in the present study could not be conclusively evaluated.

This study also had several limitations. First, the study had a small sample size, as samples were collected at regular intervals from patients in the ICU during the early stages of the COVID-19 pandemic in consideration of prognostic differences as well as sex and age similarities, making the samples valuable. This prompts concerns regarding the extent to which our findings can be generalized to a wider population. Another significant limitation of the study was that the control group consisted of patients infected with COVID-19 who were admitted to the ICU and subsequently discharged after recovery (HD group) rather than healthy individuals because of the constraints in collecting compatible samples. Additionally, although age, sex, and the duration of hospital (ICU) stay were included in the study, the small sample size restricted further investigation of their potential correlations with temporal or prognostic serum metabolite changes. Lastly, a notable limitation of the study was the lack of comprehensive data on patients’ chronic conditions, co-morbidities, and specific treatment regimens, as well as the inability to assess their potential impact on metabolite profiles. Therefore, future studies should be conducted with larger sample sizes and should incorporate comprehensive clinical characterizations including patient comorbidities, specific treatments, and the use of particular medications, thereby enhancing the robustness and predictive power of metabolomic datasets in assessing COVID-19 outcomes.

## Conclusions

COVID-19 can induce distinct metabolic signatures in the serum of patients in the ICU, facilitating patient stratification and management based on prognostic differences. Elevated serum concentrations of creatine and tyrosine were identified as potential prognostic biomarkers in the PS and Exitus groups respectively compared with the HD group, offering valuable insights into disease progression in patients in ICU without the influence of vaccination. Although our preliminary study did not define significant real-time metabolic alterations in the samples, the potential to identify temporal markers of disease progression could be likely with a larger sample size. Moreover, the most significantly impacted pathway across different prognostic groups (p < 0.05) was ‘phenylalanine, tyrosine, and tryptophan biosynthesis’. Additionally, this study indicated that several other metabolic pathways, including ‘glycine, serine, and threonine metabolism’, ‘phenylalanine metabolism’, ‘arginine and proline metabolism’, and ‘tyrosine metabolism’ were notably altered across different prognostic groups, influenced by changes in creatine or tyrosine levels. Therefore, further validation of these findings in larger-scale studies is essential to enhance disease stratification and improve patient outcomes, especially for patients admitted to the ICU for critical COVID-19.
